# Modern Sociology

**Published:** 1899-01-07

**Authors:** 


### MODERN SOCIOLOGY.

### THE LIQUOR TRADE IN AMERICA.

### IV.—HIGH LICENCE AND PROHIBITION.

It is well known that some States of the American Union absolutely prohibit the sale of intoxicating liquors. Some people say that the chief result of this is to make the keeping of drinking shops a more disreputable thing than it would otherwise be, and to let liquor of worse quality than would otherwise be permitted to be sold, be palmed off on those who demand it. They are breaking the law in drinking at all, and must take what they get. It is said, moreover, that in spite of the law a man who wants drink has no difficulty in getting it, such as it is. Of course, alcohol cannot be altogether eliminated even from a prohibition State. Druggists are permitted to keep it, and to sell it for medicinal purposes, but only on receipt of a doctor's prescription; nor are they allowed to give liquor more than once without a renewal of the prescription. Alcohol is also permitted to be kept for mechanical and manufacturing purposes. In the State of Maine, where a prohibition law is in force, the Governor has power to appoint a commissioner to furnish municipal officers of towns and cities with pure, unadulterated intoxicating liquors to be sold for medicinal, mechanical, and manufacturing purposes only. The commissioner receives from the State a salary of £300 per annum, in addition to the expenses of his office, and interest on the capital invested in his stock. The town agents who are thus supplied by the commissioner are forbidden to sell to minors without a written authorisation from parent, master, or guardian; to Indians, soldiers, drunkards, intoxicated persons; to any intemperate person of whose habits he has been notified by relations or officers of the town; to insane or incompetent persons, spendthrifts, and convicts. Any infringement of this statute is punished by a fine of not less than £2, and not more than £4. It is forbidden even to give liquor gratis to an Indian, under a penalty of £1 to £4. So severe is the law, that a fine of from £10 to £20 is inflicted on an employé of any railway or express company who brings liquor into the State with intent to sell, and the company itself is subjected to a fine of £20.

In Iowa the law is much the same; but it is puzzling to find, in face of the various statutes forbidding entirely the manufacture or sale of liquor of any kind, others which seem merely to limit the sale within certain limits and under certain circumstances. Art. 1, section 26, of the constitution says: "The manufacture and sale of any intoxicating liquor, or keeping the same for sale as a beverage, prohibited"; and we find fines, beginning at £40, and rising for subsequent offences up to £200, are to be inflicted for any infringement of this law. But, with a general prohibition like this, why should it be necessary to specify that a fine of £1 to £10 will be incurred by anyone selling liquor within 160 rods of the enclosure where an agricultural fair is being held? There are fines of various amounts for selling liquor within a mile of a polling station on the day of an election, or within three miles of a State agricultural college or farm, or within a mile of a religious field or woodland meeting, and, as in Maine, there is a fine for giving drink to an Indian, or anyone who is intoxicated. Now these detailed regulations are in themselves so wholesome and reasonable that no one would object to them. But the question is, How is there room for them, if prohibition is a reality and not a sham? Does the greater not include the less in Iowa? Can general prohibition co-exist with special permission to sell liquor?

This seems to be practically the case in Iowa; for we find in the statutes of 1894 that the licence fee is to be £120, and that no one shall obtain a licence unless the written consent of all freeholders owning property within 50 ft. of the proposed drink shop be obtained. This is just. It is hard for the owners of property to have their property go down in value because of the proximity of a public-house, from the existence of which they derive no profit whatever. It is also enjoined that no place for the sale of drink be established within 300 ft. of a church or schoolhouse. The other regulations concerning the conduct of the place are very reasonable. One of them, which obtains practically throughout the whole of the States, we would gladly see adopted here, viz., that no woman be employed on the premises. To say the least of it, the selling of intoxicants, and exchanging senseless "chaff" with those who buy it, is hardly an elevating employment for a woman. It is also ordained that no publican may sell drink to a person whose wife, husband, parent, child, brother, sister, ward over fourteen years, or employer shall forbid such sale by written notice.

The constitution of the State of Kentucky is founded on local option. Elections are to be held "in any county, city, town, district, or precinct for the purpose of taking the sense of the people upon the proposition whether or not liquor shall be sold or disposed of." If the people decide against the sale of drink, it is thereupon prohibited; if otherwise, a moderate licence fee is imposed, varying from £10 for selling malt liquors only, to £30 for the privilege of dealing in beer, wine, and spirits.

In Louisiana high licence is the rule. The price of the licence is regulated by the gross annual receipts of the house. Where the receipts are between £600 and £1,000 the licence fee is £20, and rises in steady ratio till we find houses whose receipts amount to £10,000 paying a licence fee of £300. Brewers and distillers also pay a heavy licence, assessed on the same principle. Where the annual gross receipts are less than £4,000 the fee is only £5; but it rises till businesses whose gross receipts amount to £500,000 per annum pay a licence fee of £700. In Maryland a similar method prevails; but it is based, not on the earnings of the shop, but on the rent and on the value of the stock-in-trade. Where the stock does not exceed £100 in value the licence is £3 10s.; as it rises the fee rises too, and reaches its ultimatum, where the stock is valued at £6,000, at £30. A fee of £5 is charged on a rental of £20, and is graded up till, at £2,000, the fee is £90.

Altogether, the United States seem to have tackled the drink problem with more vigour and earnestness than we have yet done. So far their progress is experimental, and perhaps it is too soon to pronounce either in favour or against any of the methods they have tried; but there is no doubt that the very experiments show a feeling on the subject deeper and stronger than any that is common in this country.

